# Supplementary material for: Structural insight into toxin secretion by contact-dependent growth inhibition transporters
Source: eLife. 2020 Oct 22;9:e58100. doi: 10.7554/eLife.58100 (PMC7644211; doi:10.7554/eLife.58100)
Supplement: Figure 2—source data 1. — List of amino acid interactions between β-barrel-helix H1, and helix H1-loop 6 in the crystal structures of CdiB A.baumannii (left) and CdiB E. coli (right). H-bond and salt-bridge interactions are labeled ‘h ‘and ‘s‘, respectively. *Interaction networks between R10-Q14 and Q214-K440 from CdiBEc are also depicted in Figure 1—figure supplement 1. [file elife-58100-fig2-data1.docx]

| CdiB *A.baumannii* | | | | |  | CdiB *E.coli* | | | | |
| --- | --- | --- | --- | --- | --- | --- | --- | --- | --- | --- |
| **β-Barrel** |  | **H1** |  | **loop6** |  | **β-Barrel** |  | **H1** |  | **loop6** |
| R356 | s | D5 |  |  |  | Q214 | h | R10^*^ | h | K440 |
| Q295 | h | V6 |  |  |  |  |  | R10 | h | S429 |
| Q295 | h | V6 |  |  |  |  |  | R10 | h | G431 |
| S266 | h | S10 |  |  |  | Q214 | h | Q14^*^ | h | G431 |
| N268 | h | S10 |  |  |  | R324 | h | Q16 |  |  |
| Y291 | h | S10 |  |  |  |  |  | Q17 | h | S429 |
| S312 | h | Q11 |  |  |  | T352 | h | Q27 |  |  |
| S270 | h | Q14 |  |  |  | N363 | h^2^ | Q27 |  |  |
| N289 | h | Q14 |  |  |  | S398 | h | R28 |  |  |
| N237 | h | R17 |  |  |  | Q400 | h | R28 |  |  |
| S270 | h | R17 |  |  |  |  |  |  |  |  |
| Q276 | h^x2^ | Q26 |  |  |  |  |  |  |  |  |
| R422 | s^x2^ | D27 |  |  |  |  |  |  |  |  |
