# Supplementary figures and images for: Structural insight into toxin secretion by contact-dependent growth inhibition transporters
Source: eLife. 2020 Oct 22;9:e58100. doi: 10.7554/eLife.58100 (PMC7644211; doi:10.7554/eLife.58100)

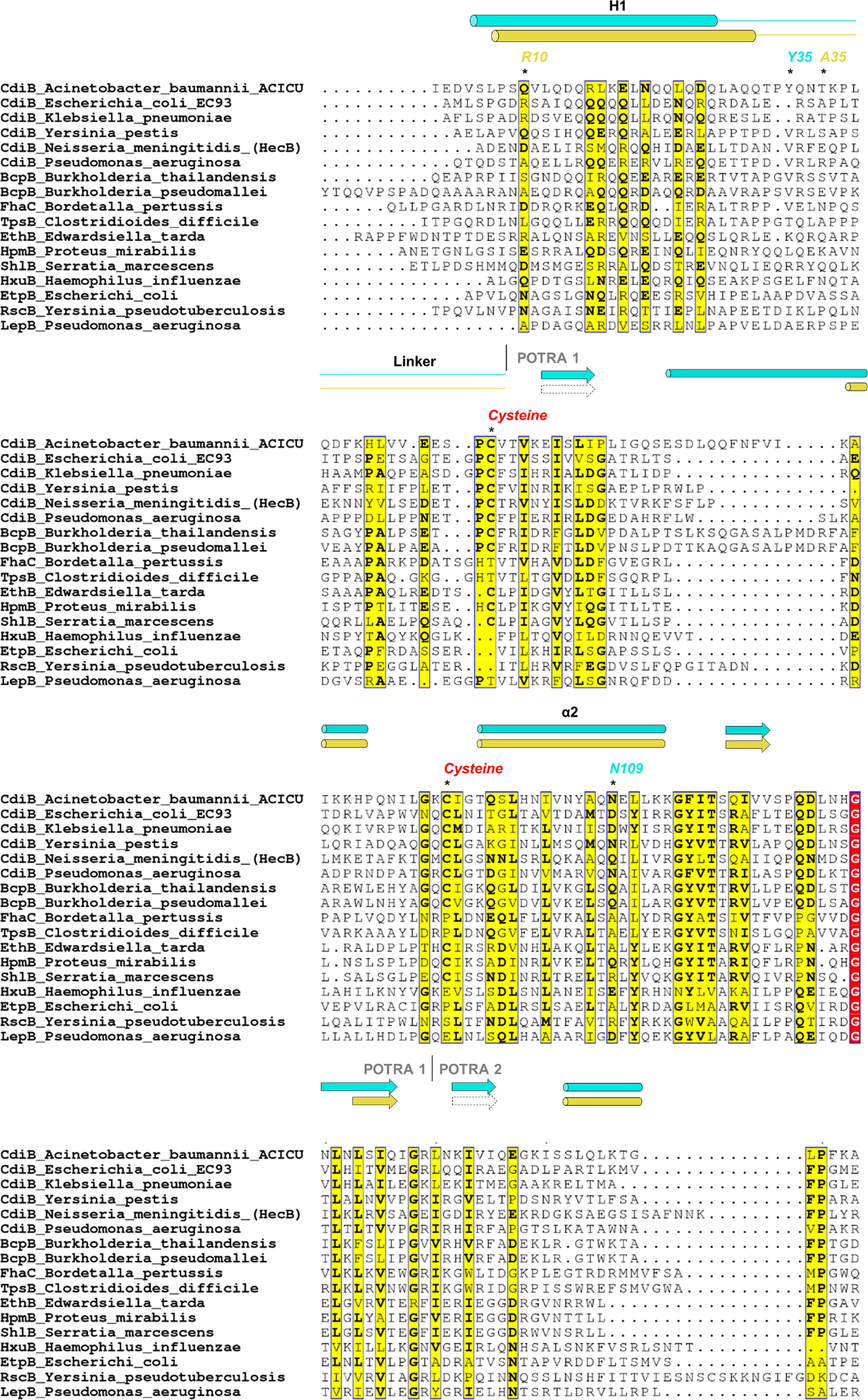


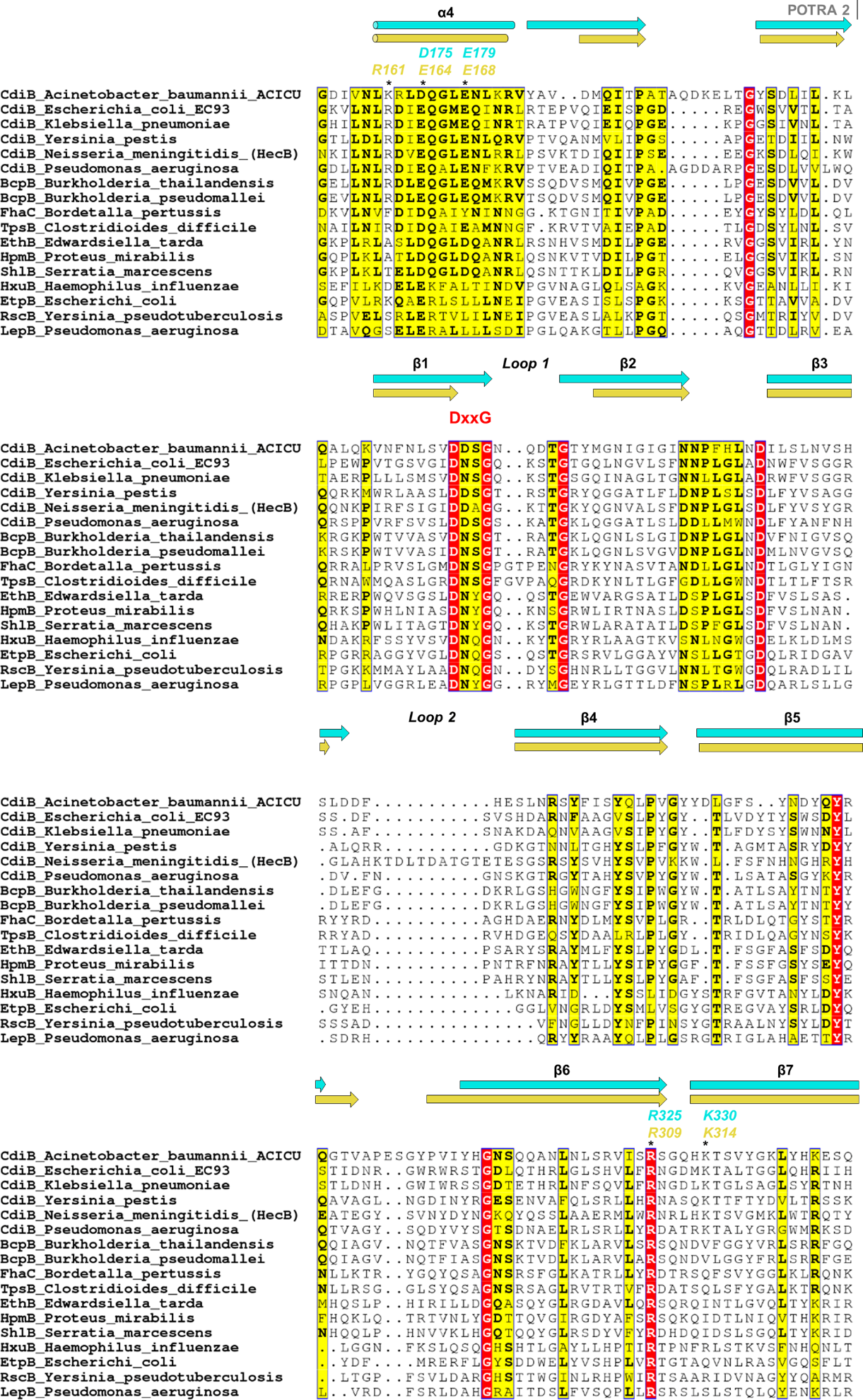


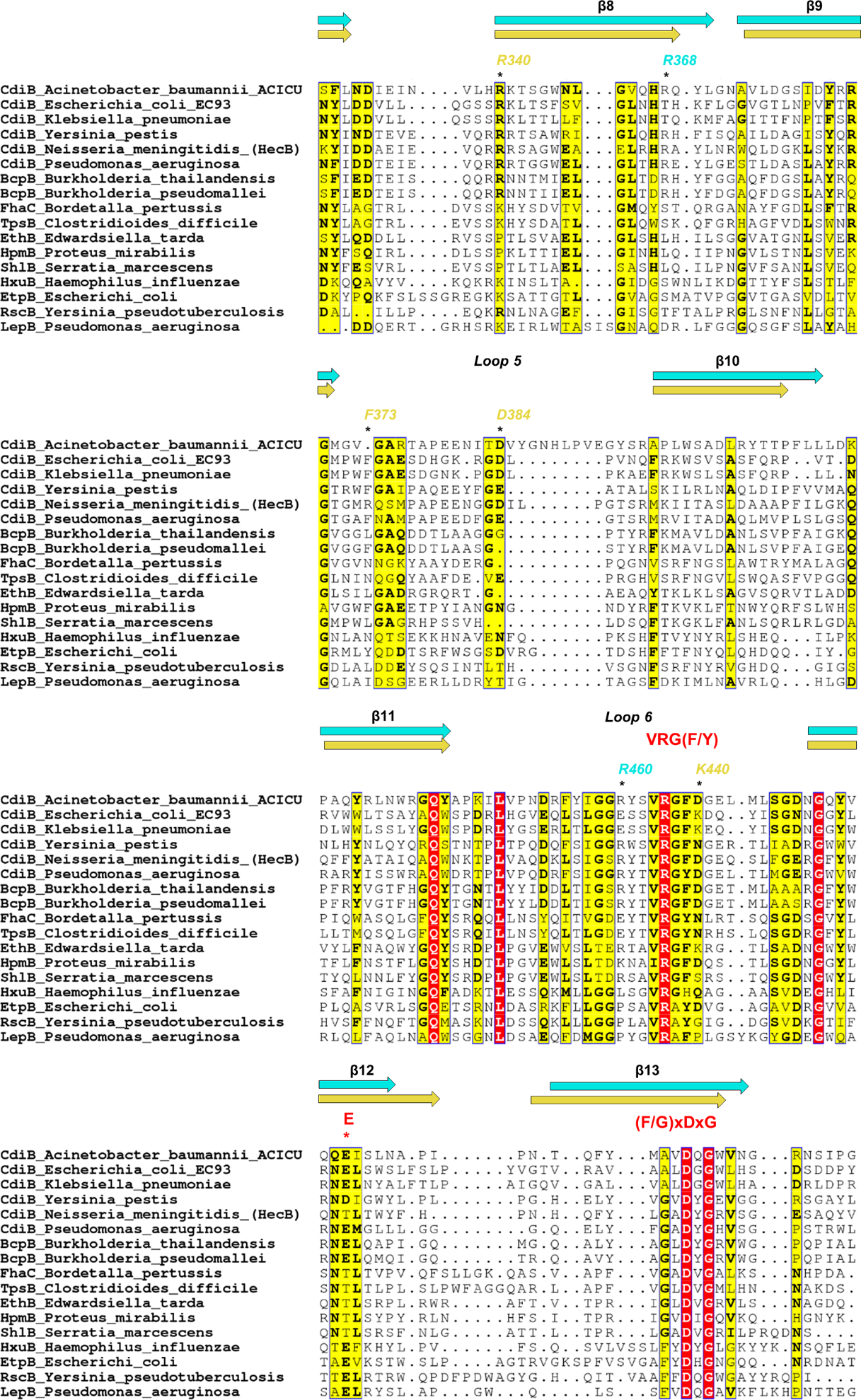


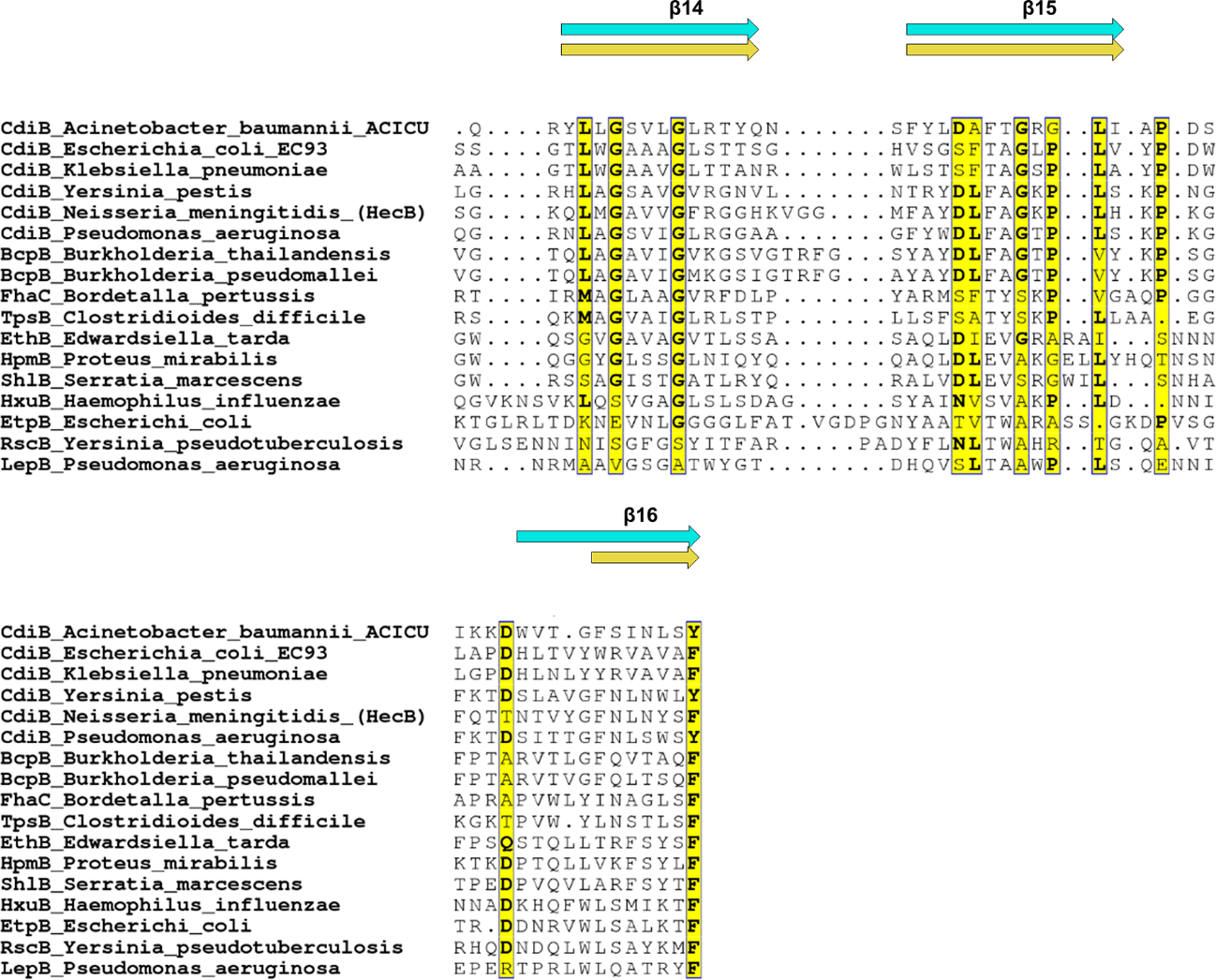

Supplement: Supplementary file 1. — The first two sequences correspond to CdiBAb and CdiBEc studied in this paper, where the names of strains are indicated. Amino acid sequences from CdiB to BcpB correspond to proteins involved in CDI mechanisms, whereas the last nine sequences (FhaC to LepB) represent other TpsB transporters. α-helices and β-strands are shown by cylinders and arrows respectively, from the structures of CdiBAb (light teal) and CdiBEc (pale yellow). The boundaries of POTRA domains are indicated in gray. Structural elements discussed in the text and figures are indicated in bold black, and important residues are marked with a star and label, in light teal for CdiBAb and pale yellow for CdiBEc. Omp85/TpsB conserved residues on L6, β13, β1 and cysteines (involved in disulfide bond formation) are shown in red. [file elife-58100-supp1.docx]
